# Biosynthesized Silver Nanoparticles and Their Antidiabetic Potential

**DOI:** 10.3390/ph18091412

**Published:** 2025-09-19

**Authors:** Angélica Sofía González-Garibay, Omar Ricardo Torres-González, Iván Moisés Sánchez-Hernández, Eduardo Padilla-Camberos

**Affiliations:** Medical and Pharmaceutical Biotechnology Unit, Center for Research and Assistance in Technology and Design of the State of Jalisco, A.C. (CIATEJ). Av. Normalistas No. 800 Col. Colinas de la Normal, Guadalajara C.P. 44270, Jalisco, Mexico

**Keywords:** green synthesis, metallic nanoparticles, diabetes, hypoglycemic

## Abstract

**Background/Objectives**: Recent advances in nanotechnology have enabled the use of biosynthesized silver nanoparticles (AgNPs) in healthcare, including the management of diabetes mellitus, a metabolic disorder characterized by impaired glucose homeostasis. AgNPs have shown promising effects on enzymes, insulin signaling, gut hormones, and in vivo models. Despite the availability of oral treatments, challenges persist, prompting interest in novel therapies such as AgNPs, which are currently under investigation in various in vitro and in vivo studies. **Methods**: This narrative review was conducted through a PubMed search using the terms “antidiabetic + activity + AgNPs” in April 2025. Relevant articles published in English were selected and analyzed, with emphasis on studies employing biosynthesized AgNPs from plants in in vitro and in vivo models. Information was extracted regarding the experimental approaches used to evaluate antidiabetic activity, the plant sources employed, nanoparticle characteristics, concentrations tested, and corresponding outcomes. **Results**: The biosynthesis of AgNPs employs bioactive compounds from plants, making it an environmentally friendly green synthesis method. Plant extracts are the most common biomaterial for AgNPs biosynthesis. Most of the in vitro studies evaluated the inhibitory effect of AgNPs on α-glucosidase or α-amylase; meanwhile, in animal studies, the main parameter evaluated is blood glucose level. **Conclusions**: The antidiabetic potential of AgNPs is becoming increasingly evident as ongoing research continues to explore their effects through both in vitro and in vivo studies. In this review, the current state of research regarding the potential use of AgNPs for diabetes management and treatment is presented, highlighting recent findings and discussing future perspectives in the field.

## 1. Introduction

Diabetes mellitus (DM) is a group of metabolic disorders of carbohydrate metabolism in which glucose is both underutilized as an energy source and overproduced due to inappropriate gluconeogenesis and glycogenolysis, resulting in hyperglycemia [1,2]. DM is conventionally classified into several clinical categories: type 1 diabetes, type 2 diabetes (T2D), specific types of diabetes due to other causes, and gestational diabetes. Type 1 diabetes is due to autoimmune β-cell destruction, usually leading to absolute insulin deficiency. T2D is due to a progressive non-autoimmune loss of adequate β-cell insulin secretion, frequently with a background of insulin resistance and metabolic syndrome. Specific types of diabetes due to other causes, such as genetics or other organ-related problems, are rare. Gestational diabetes is diagnosed in the second or third trimester of pregnancy [1]. Some of the causes of DM include aging, a rapid increase in urbanization, and obesogenic environments [3].

According to the International Diabetes Federation (IDF), the current global prevalence of DM among adults aged 20–79 years is estimated at 11.1%, affecting approximately 588.7 million people in 2024. According to estimates, this figure is projected to rise to 13% by 2050, impacting around 852.5 million people. The IDF recently estimated that the total global healthcare expenditure attributable to diabetes among adults aged 20–79 years was USD 1,015 billion in 2024, with projections indicating an increase to USD 1,045 billion by 2050. Noticeably, approximately 90% of the prevalence of DM corresponds to people with T2D [3].

T2D is a disorder characterized by an impaired glucose homeostasis due to a dysregulation of carbohydrate, lipid, and protein metabolism. T2D is caused by a combination of genetic and environmental factors. However, impaired insulin secretion, insulin resistance, or a combination of both is considered the core pathophysiological defect. A T2D diagnosis is based on measurements of fasting plasma glucose, 2 h plasma glucose after an oral glucose tolerance test, and glycosylated hemoglobin (HbA1c) [4,5,6,7]. Symptoms and signs include high blood glucose levels over a prolonged period, frequent urination, increased thirst, and elevated hunger.

Prolonged and untreated DM can lead to serious complications on the cardiovascular system, kidneys and eyes, some of which are life-threatening [5,7,8]. T2D has long been identified as an incurable chronic disease. The best outcome that can be expected is the amelioration of symptoms. Approximately 50% of T2D patients will need insulin therapy within ten years of diagnosis. Although, in the past, diabetes was called chronic and irreversible, this paradigm has now changed [9]. Management for glucose control and diabetes remission includes modifications in eating and lifestyle habits [10,11]. However, sustained weight loss maintenance and complete adherence to diet and physical activity recommendations are uncommon among individuals with T2D [12].

The current therapeutics for the management of type 2 diabetes (T2D) include medications prescribed either in the presence or absence of exogenous insulin. Based on their mechanisms of action, these agents are broadly classified into four major classes: (a) insulin sensitizers, such as biguanides and thiazolidinediones; (b) insulin secretagogues, including sulfonylureas and glinides; (c) α-glucosidase inhibitors, such as acarbose and miglitol, which delay carbohydrate absorption; and (d) other agents, such as sodium–glucose co-transporter-2 (SGLT2) inhibitors and bile acid sequestrants. Some of these drugs carry the risk of side effects, including hypoglycemia, weight gain, and cardiovascular complications. However, some new drugs have been associated with weight loss [2,13,14,15].

In addition, one disadvantage of the current oral treatments of T2D is related to their low bioavailability and the immediate release of the drug into the bloodstream. In this context, nanotechnology, including different nanosystems and nanomaterials, is also applied for drug delivery, diagnostics, and tissue repair [15]. Based on the type of nanomaterial used in their composition, nanoformulations targeting T2D can be classified into polymer-based, lipid-based, and inorganic nanoformulations [16]. Inorganic nanoformulations can include the biosynthesis of silver nanoparticles (AgNPs), which can be carried out using whole plants or their parts [17].

Different parts of plants contain bioactive compounds, such as flavonoids and alkaloids, that can be used for diabetes treatment. Various techniques can be applied for their extraction. Their mechanisms of action involve improving glucose metabolism, exerting a hypolipidemic effect, enhancing the pancreatic effect, providing antioxidative benefits, managing diabetes-related complications, and exhibiting insulin-like activity [18]. According to ethnobotanical information reports, approximately 800 plants may possess antidiabetic effects [19].

Various approaches to the synthesis of metallic nanoparticles are available. These reactions have common steps: reduction, nucleation, growth, coarsening, and/or agglomeration. One widely used method involves reducing metal salts in an aqueous or organic solvent [20]. Research on AgNPs’ antidiabetic potential has focused primarily on inhibition studies of enzymes involved in carbohydrate metabolism and on animal models with drug-induced diabetes. The biosynthesis of AgNPs using parts of plants could be used for medical applications with less toxicity [21,22].

Recent studies have explored the potential application of AgNPs in the management of endocrine disorders such as DM, a prevalent metabolic disorder affecting millions worldwide. Given the global impact of DM, there is significant scientific interest in developing innovative strategies for its effective control [3,23].

An overview of existing research was conducted in PubMed in April 2025 using the search term “antidiabetic + activity + AgNPs”. Relevant articles published in English were selected and reviewed, focusing on the utilization of biosynthesized AgNPs using plants in in vitro and in vivo studies. Information regarding the methods used to evaluate the antidiabetic effects of AgNPs, the plant sources employed, the nanoparticles’ characteristics, the concentrations tested, and the corresponding results was extracted from the collected data.

In this review, the current state of research regarding the potential use of AgNPs for diabetes management and treatment is presented, highlighting recent findings and discussing future perspectives.

## 2. Biosynthesis of AgNPs

Nanotechnology deals with the design, fabrication, and application of small structures or small-sized materials. The size of nanoparticles spans from subnanometers to several hundred nanometers, where one nanometer is one billionth of a meter (1 × 10^−9^) [24]. Nanotechnology has advanced significantly in recent decades due to an improved understanding of nanostructure growth processes, as well as enhanced methods for analysis and characterization. These developments have enabled a wide range of applications across fields such as agriculture, textiles, environmental science, and medicine. Nanotechnology applied in medicine, often referred to as nanobiomedicine, has contributed to advancements in diagnostics, treatment, screening, disease prevention, and proactive healthcare [25].

The characteristics of nanoparticles, including size, shape, and the orientation of surface functional groups, are highly relevant. However, one of their most defining features is their high surface area-to-volume ratio, which facilitates their infiltration into biological tissues and fluids. This property enables processes such as endocytosis, distribution, retention, and elimination within biological systems [26].

Metallic nanoparticles exist in nature, and several historical objects, such as the pigments used by the Mayans in Chichen Itzá have been shown to contain iron and chromium nanoparticles [27]. In the 19th century, metal nanoparticles were used in photochemistry and photography and in glass molding [28]. However, current research has focused on developing different synthesis routes and evaluating their properties [29].

**Figure 1:**
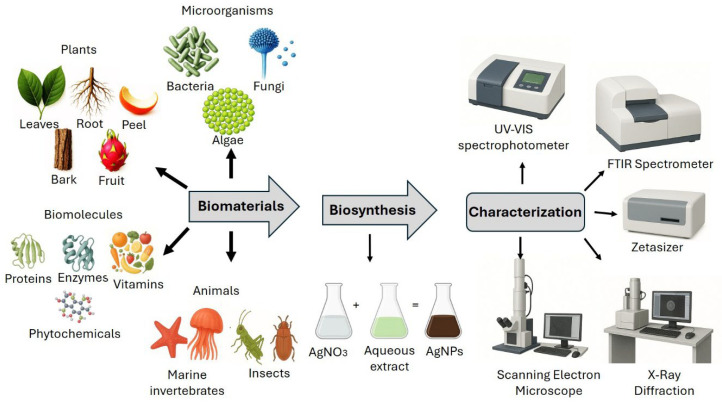
Biosynthesis and characterization of AgNPs.

The synthesis of nanoparticles can be carried out using physical, chemical, and biological methods. The main disadvantage of physical and chemical methods is that they have a high cost, both in economic and environmental terms, because they involve the use of dangerous or toxic substances [30]. Therefore, recent research has focused on developing clean nanoparticle synthesis methods that do not use risky substances and are environmentally friendly; this can be achieved using biological systems with different biomaterials, such as microorganisms and plants [31,32,33,34]. Regarding plants, their seeds, leaves, fruits, flowers, peel, roots, stems, bark, and food by-products have been used; an extract is made, usually aqueous, and serves as a reducing agent for silver nitrate (silver salt) [21,22,35]. Bacteria, fungi, algae [17,36], biomolecules, and even animal parts [37,38] have also been used for the biosynthesis of AgNPs. Thus, the sources for the elaboration of AgNPs are broad (see Table 1 and Figure 1).

The synthesis of AgNPs can be influenced by different parameters, such as temperature, pH, concentration of reaction components, reducing or stabilizing agents, and the molar ratio of the surfactant/stabilizer and precursor [20]. Notably, the phytochemicals such as alkaloids, phenolics, terpenes, saponins, and steroids present in plant extracts act as reducing, capping, and stabilizing agents, facilitating the bioreduction of Ag^+^ ions to Ag^0^, and leading to the formation of AgNPs [39].

Although nanoparticles can be synthesized from different metals, AgNPs represent a clear example of the multiple applications that these materials can have, since they have been used in clothing, the food industry, electronics, biosensing, environmental remediation, medical healthcare, and consumer products [40].

**Table 1:** Examples of biomaterials for the biosynthesis of silver nanoparticles.

Biomaterial		Silver Nanoparticle Size	Reference
**Plants**	**Scientific name**		
Leaves	*Theobroma Cacao Linneu*	10.3 nm	[41]
Fruit	*Aegle marmelos*	159–181 nm	[42]
Bark	*Heritiera fomes* and *Sonneratia apetala*	20–30 nm	[43]
Root	*Coleus forskohlii*	5–35 nm	[44]
Peel	*Stenocereus queretaroensis*	98.9 nm	[45]
**Microorganisms**			
Bacteria	*Bacillus cereus*	5–7.06 nm	[46]
Fungi	*Aspergillus* *sydowii*	1–24 nm	[47]
Algae	*Asterarcys sp.* microalga	35–52 nm	[48]
**Biomolecules**			
Enzymes	Nitrate reductase from *Fusarium oxysporum*	50 nm	[49]
Vitamins	Vitamin C	26.5 nm	[50]
Proteins	Bovine serum albumin, lysozyme, among others	Non determined	[51]
Phytochemicals	Naringenin	10–21 nm	[52]
**Animals**			
Marine invertebrates	*Marphysa moribidii*	40.19 nm average	[53]
Insects	*Mang mao* wings	40–60 nm	[37]

## 3. Characterization of AgNPs

The characterization of biosynthesized AgNPs is carried out using different methodologies, such as spectrophotometry; the surface plasmon resonance of silver ions has been detected at wavelengths of 400–460 nanometers [54,55]. X-ray diffraction analysis is used to determine the crystalline nature of AgNPs. Additionally, Fourier transform infrared spectroscopy measurements are used to determine the interactions of silver ions with the phytochemical compounds responsible for stabilizing the nanoparticles. The size and morphology of AgNPs are evaluated using scanning and transmission electron microscopy coupled with energy-dispersive X-ray spectroscopy, whereas size distribution and stability can be determined using dynamic light scattering spectroscopy [54,56,57,58]. The chemical composition of biosynthesized AgNPs can be analyzed by utilizing scanning electron microscopy coupled with energy-dispersive X-ray spectroscopy [57]. On the other hand, evaluating the zeta potential of a colloidal dispersion is crucial for assessing its stability and resistance to agglomeration [59].

## 4. Metabolism of AgNPs

Nanoparticles, ranging in size from 1 to 100 nm, have a high surface area-to-volume ratio, making them highly reactive and capable of penetrating body tissues and fluids. Their size and surface properties play a key role in endocytosis, distribution, accumulation within biological systems, and eventual elimination [26]. Due to their size, most nanoparticles do not reach their intended target and are instead sequestered by the liver and spleen, if larger than 6 nm, or eliminated through the kidneys, if smaller than 6 nm, after their administration into the body. While the route of administration influences nanoparticle processing, the liver acts as a primary biological filter, sequestering 30–99% of administered nanoparticles from the bloodstream. The development of solutions that overcome the liver will be key to enabling the use of nanoparticles for medical applications [60] when the liver is not the intended target.

The main applications of AgNPs in the biomedical area include therapeutic applications such as antioxidants, diagnosis, biomolecular detection, and controlled drug release, among others [61,62].

In vitro studies have demonstrated that the dose, size, and coating of AgNPs affect their cellular uptake; meanwhile, in vivo distribution studies have reported silver accumulation and toxicity to both local and distant organs [63].

Accumulation occurs in the liver, kidneys, spleen, brain, lungs, and testicles. Nanoparticles can persist in the body for several weeks after the end of oral exposure, particularly in the brain and testicles [64].

## 5. Toxicology of AgNPs

Regarding the potential toxicological risks of nanoparticles, the increasing biomedical and commercial applications of AgNPs have raised concerns about their interactions with the environment and human health [65]. The potential toxicity of silver nanoparticles is influenced by factors such as particle size, shape, concentration, and duration of exposure. However, their safety remains a matter of debate, underscoring the need for comprehensive toxicity assessments and well-designed clinical trials to establish the safety profile of AgNPs in diabetes treatment [57]. Furthermore, global harmonization of nanomaterial regulations and standa rdized toxicological testing protocols is essential to ensure the safe use of nanomaterials for manufacturers, healthcare professionals, and the general public.

Recently, an in vitro study provided a first attempt to calculate the human effect factors (HEF) value for nanoparticles, including AgNPs, which can be used to establish their impact category in a life cycle assessment. The non-carcinogenic HEF (case/kg intake) for AgNPs was 5.9 × 10^−1^, compared with 7.5 × 10^−3^ for CuO-nanoparticles, and 2.5 × 10^−2^ for zinc-nanoparticles [65]. Although the dose of nanoparticles in animal models varies, one study in rats estimated a no observable adverse effect level (NOAEL) of 30 mg/kg and a lowest observable adverse effect level (LOAEL) of 125 mg/kg [66]. Nevertheless, providing general warnings remains challenging, as data in the literature must take into account the type and surface charge of nanoparticles, their particle aggregation, cell lines, experimental designs, and various observational endpoints [67,68].

## 6. Mechanisms of Action of AgNPs in Type 2 Diabetes Mellitus

T2D risk factors include a complex combination of genetic, metabolic, and environmental factors; the main modifiable risk factors are obesity, low physical activity, and an unhealthy diet. The pathophysiological changes are characterized by β-cell dysfunction, insulin resistance, and chronic inflammation leading to hyperglycemia [4,69].

Overconsumption of food leads to obesity and is associated with gluco-lipotoxicity; it also stimulates insulin secretion and the production of reactive oxygen species, which in turn leads to an abnormal generation of inflammatory molecules. This pro-inflammatory environment causes insulin resistance in muscle, liver, and adipose tissue; as a consequence, β-cells continue the overproduction of insulin to control hyperglycemia until dysfunction of the pancreas occurs. However, other processes and behavioral risks are involved, including aging, resistance and/or deficiency of incretin hormones such as GLP-1 and gastric inhibitory polypeptide (GIP), hypersecretion of islet amyloid polypeptide, smoking, and sleep habits [4,69]. Also, gut microbiota dysbiosis has been reported to play a role in driving T2D [69,70].

On the other hand, sustained hyperglycemia induces the formation of advanced glycation end-products (AGEs), which worsen oxidative stress and non-enzymatic glycation in proteins such as hemoglobin [69]. Modulating oxidative stress holds significant therapeutic potential in the treatment of diabetes [71]. Exploring the antioxidant benefits of AgNPs along with their antidiabetic effect has also been investigated [72,73] using different in vitro methods (Table 2) and animal models.

The antidiabetic activity of AgNPs has been primarily evaluated in murine models, commonly induced by alloxan or streptozotocin, both of which are well known for damaging the pancreas and inducing diabetes (Table 3).

An effective strategy for lowering elevated blood glucose levels is enzyme inhibition. α-amylase hydrolyzes starch and releases maltose and a range of branched-oligosaccharides that are further hydrolyzed by α-glucosidase into absorbable monosaccharides such as glucose. Extensive research has focused on identifying compounds that target these enzymes to regulate blood sugar levels [117]. Acarbose [118], voglibose, and miglitol are three inhibitors of these two enzymes [119]. This inhibition decreases monosaccharide cleavage from complex carbohydrates in the diet, improving hyperglycemia. The side effects of some of these drugs, such as flatulence, bloating, and diarrhea, highlight the need for the development of new inhibitors [118,119], specifically ones with stronger inhibition against α-glucosidase and a lower one against α-amylase [117]. Many studies have evaluated the inhibitory effect of AgNPs on α-glucosidase and α-amylase in vitro (Table 4). While certain studies have reported a higher inhibitory activity against α-amylase [73,74,75,80,99], others have observed a more pronounced effect on α-glucosidase [72,103]. The size of SAgNPs varies among studies, and smaller sizes appear to be associated with more efficient inhibitory activity. For example, *Leucosidea sericea* (2.9–7.8 nm) demonstrated α-glucosidase inhibition with IC_50_ values ranging from 8.75 to 21.48 μg/mL [120]. Similarly, *Berberis lyceum* (11.02 nm) exhibited inhibitory activity comparable to acarbose [103], while *Cleome viscosa* (5–50 nm) showed dual inhibitory activity against both α-amylase and α-glucosidase [85].

After ingestion of a meal, insulin is released and the secretion of incretin hormones, such as glucagon-like peptide-1 (GLP-1) and gastric inhibitory polypeptide (GIP), is increased. GLP-1 and GIP are gut-derived hormones that enhance insulin secretion, suppress glucagon release, and delay gastric emptying. These incretin hormones play a critical role in modulating glucose metabolism and reducing blood glucose levels [151]. However, the enzyme dipeptidyl peptidase-4 (DPP-4) promotes the degradation of both GLP-1 and GIP. Inhibition of DPP-4 activity results in elevated levels of these incretin hormones, thereby enhancing insulin secretion and suppressing glucagon release, ultimately contributing to improved glycemic control. DPP-4 inhibitors such as sitagliptin, vildagliptin, saxagliptin, linagliptin, and gemigliptin have been developed for clinical use [152]. AgNPs have also been evaluated as potential DPP-4 inhibitors [83]. It is worth mentioning that natural products have also been identified as DPP-4 inhibitors [151].

One approach to treating diabetic patients involves stimulating insulin secretion using thiazolidinedione drugs. Another strategy targets glucagon and related molecules, as glucagon promotes glucose production. These mechanisms have been investigated using AgNPs in in vitro models with pancreatic and liver hepatic cell lines [87].

In vitro studies have employed glucose dialysis retardation assays and glucose adsorption capacity to evaluate the potential of bioactive compounds in modulating glucose absorption. The glucose dialysis retardation assay suggests that certain compounds may delay glucose absorption in the intestine and prevent postprandial hyperglycemia [74]. Similarly, the glucose adsorption assay demonstrates that some compounds can effectively bind to glucose, reducing its availability for absorption. This mechanism has been further supported by studies in yeast models, where reduced facilitated glucose diffusion was observed [89]. Carbohydrate-rich foods are classified based on their glycemic index (GI), which represents their ability to increase blood glucose levels. Foods categorized as high GI cause a more rapid and bigger increase in blood glucose compared to medium-or low-GI foods. In addition to GI, the glycemic load (GL) is another measure used to classify foods, incorporating both the GI and the quantity of carbohydrates consumed in a meal. Foods are categorized as low-, medium-, or high-GL. Numerous studies have demonstrated the beneficial effects of dietary interventions with low-GI or low-GL foods in the management of diabetes [153]. Bioactive compounds capable of modulating GI or GL may serve as potential therapeutic agents for diabetes management.

Tyrosine protein phosphatase non-receptor type 1 (PTP1B; also known as protein tyrosine phosphatase 1B) downregulates insulin signaling through changes to insulin receptor activity. Although the physiological activity of PTP1B depends on cell type, PTP1B inhibition can improve insulin sensitivity and exert benefits on DM [58,154].

Oxidative stress has been associated with T2D and is defined as a condition characterized by elevated levels of reactive oxygen and nitrogen species, together with reduced antioxidant levels. This imbalance disrupts normal cellular processes, as the high electron reactivity of these species enables their interactions with biomolecules such as lipids, proteins, and nucleic acids. Reactive species may originate from exogenous or endogenous sources, with the mitochondrial electron transport chain—particularly under nutrient overload—being a major endogenous contributor [155]. The potential antioxidant activity of AgNPs arises from phytochemicals present in plants, such as phenolics, terpenoids, and flavonoids, which enable them to neutralize reactive species [75].

Some studies have evaluated the antidiabetic effect of AgNPs not only via amylase or glucosidase inhibition, but also by measuring their radical scavenging activity using the DPPH (1,1-diphenyl-2-picrylhydrazyl) or ABTS 2,2-azino-bis (3-ethylbenz-thiazoline-6-sulfonic acid) assays [75,123]. In regard to the in vitro assays among the articles found for this review, it is noted that the lowest size of AgNPs was 2.9 nm [120]; one study reported a nanoparticle size of 170 nm [125]. In one article that synthesized many types of nanoparticles from fractions of *Rosa indica* L., one of them was reported to be as large as 770 nm [132]. Some studies have utilized diverse doses of AgNPs to evaluate α–amylase; among the doses employed, some were as low as 0.3 μg/mL [137], utilizing 1 mL of salivary α-amylase, while other studies have used doses as high as 1.6 mg/mL [121], but employed 0.05 g/100 mL α-amylase. Other findings report using 30 mg/mL of nanoparticles to evaluate α–amylase (0.4 U/mL) inhibition [143]. This variability might be related to the experimental design, mainly the enzyme activity, but also the type of starch used, pH, and temperature [156]. Enzyme concentration must be taken into account when interpreting and comparing the effects of AgNPs treatment.

The production of AGEs is associated with the pathogenic mechanisms underlying T2D, as well as the development of diabetic complications and other chronic diseases. AGEs can be assessed through inhibition assays targeting vesperlysine-like and pentosidine-like AGEs, which are naturally fluorescent and cross-linked [95]. The anti-glycation activity of AgNPs has been previously reported [129].

Recently, one study employed in silico molecular docking approaches to evaluate interactions between silver atoms and proteins such as α-amylase, α-glucosidase, insulin, and glucagon, demonstrating that silver atoms can interact with the amino acid residues of these proteins, since the binding energy resulted in <0 kcal/mol [87].

Regarding animal models, various doses and particle sizes of AgNPs have been evaluated. Sizes as small as 9 nm [110], and up to approximately 100 nm have been tested in murine and fish models [104,115]. Most AgNPs treatments were administered intraperitoneally or orally for periods ranging from 9 to 30 days, with exceptions such as *Momordica charantia*, which was administered for 14 weeks [111]. Oral doses have ranged from as low as 50 μg/kg [115] to as high as 2000 mg/kg [111], while intraperitoneal administration of 10 mg/kg has also been reported [116]. As expected, blood glucose level has been the primary parameter evaluated in in vivo models, as reported by Virgen-Ortiz (2015) and more recently by Hosen E et al. (2024) [109,110]. Most diabetic models are induced using streptozotocin rather than alloxan. Several studies have demonstrated the hypoglycemic effects of biosynthesized AgNPs in diabetic animals. For example, *Rumex hymenosepalus* reduced blood glucose levels by 50% in diabetic rats within 9 days [110]. Benefits have been observed not only in blood glucose regulation but also in tissue morphology, including that of the liver, kidneys [80,96,102,108], and even the pancreas [80,96,108].

**Figure 2:**
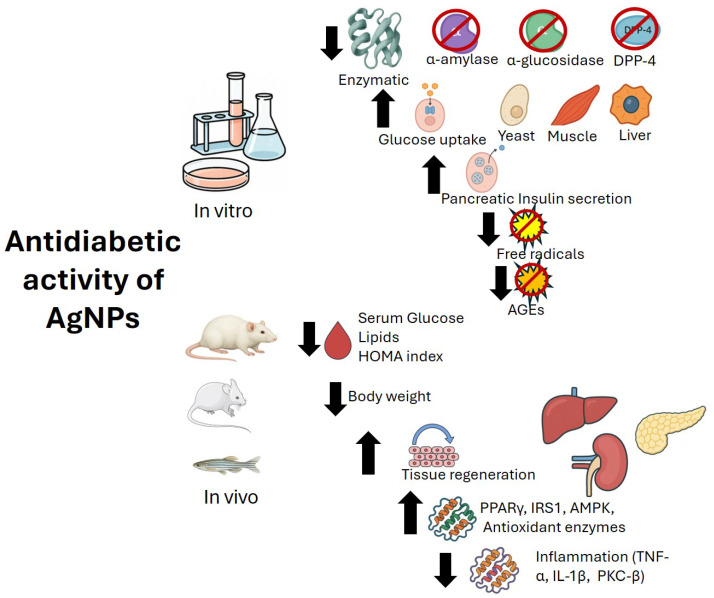
Proposed mechanisms of action of AgNPs in diabetes mellitus. AGEs: Advanced glycation end products; DPP-4: Dipeptidyl peptidase 4; AMPK: AMP-activated protein kinase; IRS1: Insulin receptor substrate 1; PPARγ: Peroxisome proliferator-activated receptor gamma; TNF-α: Tumor necrosis factor-alpha; IL-1β: Interleukin-1 beta; PKC-β: Protein kinase C-beta.

Consistent with in vitro findings on size, smaller AgNPs in animal models appear to exert more efficient antidiabetic effects. This was demonstrated with AgNPs biosynthesized from *R. hymenosepalus* (9 nm) [110], *Lawsonia inermis* (14.9 nm) [112], and *Eysenhardtia polystachya* (10–12 nm) [93], all of which lowered blood glucose and, in some cases, improved lipid profiles. Findings from animal studies suggest that AgNPs exert multitarget effects, not only reducing blood glucose but also modulating inflammatory processes and exhibiting antioxidant and regenerative activities (Table 5). The proposed mechanisms of action of AgNPs are illustrated in Figure 2.

## 7. Current Challenges in the Treatment of Type 2 Diabetes Mellitus

Some studies have shown that weight loss can produce a remission of T2D, defined as no longer meeting the diagnostic criteria for T2D. Both diet and a physically active lifestyle are the cornerstones of remission, and it is worth mentioning that long-term maintenance of weight loss is challenging [157].

Dietary restrictions are commonly prescribed to improve metabolic control in T2D. National and international guidelines for nutritional and lifestyle recommendations are available. Some recommendations focus on the consumption of low glycemic index foods to improve hyperglycemia [153,158]. Other dietetic approaches have even had an impact on the low-grade inflammation present in T2D [159]. Regular exercise is essential for the management of T2D. However, exercise training alone, without any sort of dietary intervention, may not lead to diabetes remission [157].

DM is a major public health epidemic despite recent advances in both pharmaceutical and technological treatment options [9]. The pharmaceutical industry encounters challenges in innovation, including the complexity of drug discovery, high research and development costs, risk of failure, and prolonged regulatory approval processes, among other factors [160,161]. The interest in natural products in drug development seems undeniable [161].

## 8. Study Limitations

Doses of AgNPs are reported using various units, and not all studies provide IC_50_ values for α-amylase and α-glucosidase inhibition. Many investigations rely solely on enzymatic activity to support antidiabetic effects, with relatively few animal studies compared to in vitro studies. Another limitation of studies with AgNPs is the lack of safety evaluations using appropriate methodologies to establish toxicological risk and its possible effects on the environment.

## 9. Conclusions

This review provides an extensive overview of the antidiabetic effects of AgNPs, with particular emphasis on differentiating findings from in vitro and animal studies. Such distinctions are essential for interpreting current evidence and guiding future research toward clinical translation. Most studies have focused on the enzyme inhibitory activity of AgNPs; however, a smaller number have examined glucose uptake in cells, the expression of relevant genes, and the non-enzymatic glycosylation of proteins. In addition, a few other types of studies have also been reported, although they remain limited.

The biosynthesis of AgNPs employs bioactive compounds from plants instead of hazardous chemicals, making it an environmentally friendly green synthesis method. Plant extracts are the most common biomaterial for AgNP biosynthesis. Optimization of the biosynthesis conditions is highly recommended to enhance the efficiency and reproducibility of AgNP production. However, safety concerns must still be addressed given the increasing utilization of AgNPs; this, in turn, requires further investigation into their toxicological effects. Most of the antidiabetic screening found in this review was performed in vitro using cellular and enzymatic assays and a few animal models. In addition, the shape and size of AgNPs may influence their antidiabetic efficacy and should therefore be considered in future investigations.

T2D remains a major public health challenge despite recent advances in nanobiomedicine. The antidiabetic potential of AgNPs is increasingly supported by evidence from both in vitro and in vivo studies, suggesting their promise in the development of nanoparticle-based therapies. Nevertheless, further investigations are required, particularly regarding pharmacological properties, biocompatibility, and cytotoxicity.

Additionally, although several mechanisms of action have been proposed, the precise mode of action in the altered processes in T2D remains unclear. Such studies are essential to optimize dosage, stability, and safety, thereby enabling the design of clinical trials and advancing the potential commercialization (i.e., scaling up the biosynthesis process, regulatory alignment) of AgNP-based antidiabetic agents.

## Figures and Tables

**Table 2:** In vitro methods to evaluate the antidiabetic activity of AgNPs.

Methods	Fundamentals	Reference
Inhibition of α-glucosidase or α-amylase	Enzymes involved in carbohydrate metabolism; their inhibition prevents the increase in blood glucose.	[74,75,76,77,78,79,80]
Glucose dialysis retardation index (GDRI)	A dialysis membrane is used, resembling the human intestine, where an amount of glucose is placed with the test compound, and its ability to release the glucose to an external solution is measured.	[74]
Glucose adsorption capacity	It is a colorimetric assay which evaluates the ability of a compound to bind glucose under incubation conditions at 37 °C for 6 h.	[81]
Tyrosine phosphatase 1B inhibitory assay (PTP1B)	This enzyme is a negative regulator of the insulin signaling pathway.	[82]
Dipeptidyl peptidase 4 (DPP-4) inhibition assay	Inhibition of this enzyme regulates the function of incretins, which in turn stimulate insulin production.	[83]
Non-enzymatic glycosylation of hemoglobin	High values of glycated hemoglobin have been associated with nephropathy, retinopathy and cardiovascular disease in DM.	[84,85]
Advanced glycation end-products	These molecules are a product of hyperglycemia and are involved in the complications of DM.	[86]
Glucose uptake in muscle cells	L6 cells (myoblasts of rat skeletal muscle) are the model skeletal cells to study the mechanisms of glucose uptake by muscle cells.	[87]
Glucose uptake in liver cancer cells	Hep-2 liver cancer cells	[33]
Glucose uptake in yeast cells	Glucose uptake in yeast cells depends on its diffusion through the membrane, so an increase is favorable.	[88,89,90]
Hepatic glucose production	HepG2 hepatic cells can release glucose through gluconeogenesis and glycogenolysis	[87]
Insulin secretion	Insulin secretion by MIN6 pancreatic cells	[87,88,89,90,91]
Expression of the Peroxisome proliferator-activated receptor gamma (PPARγ)	PPARγ enhances insulin sensitivity. PPARγ expression has been evaluated in RINm5F insulinoma cells	[92]
Peroxide-induced pancreatic INS-1 cell damage	H_2_O_2_ may induce damage with an increase in oxidative stress, as in diabetic chronic hyperglycemia.	[93]
Assay of non-enzymatic glycosylation of hemoglobin (HbA1c)	Hemoglobin reacts non-enzymatically with glucose through a process known as glycation, resulting in the formation of glycated hemoglobin (HbA1c).	[85,94]
Vesperlysine-like advanced glycation end products (AGEs) and pentosidine-like AGEs inhibition assays	AGEs accumulation is associated with hyperglycemia and the pathogenic mechanisms underlying T2D	[95]
1,1-diphenyl-2-picrylhydrazyl (DPPH) scavenging activity *	DPPH is a well-known free radical; its reduction would show effective scavenging activity.	[76,83,96,97,98]
2,2-azino-bis (3-ethylbenz-thiazoline- 6-sulfonic acid-) (ABTS) antioxidant assay *	ABTS is a free radical; its reduction would show effective scavenging activity.	[76,78,80,99,100]
Ferric reducing antioxidant power assay (FRAP) *	Evaluation of the ability to reduce ferric ions (Fe^3+)^ to ferrous ions (Fe^2+^)	[72]
Total reducing power assay (TRP) or reducing power test *	Calculation of the reduction power utilizing potassium ferricyanide	[72,73,100]
Nitrite/nitrate oxide (NOx) assay *	Evaluation of the potential of nitric oxide radical scavenging	[100]
Total antioxidant capacity (TAC) *	Reduction of molybdenum	[72]
Hydroxyl radical scavenging assay (HRSA) *	Scavenging of hydroxyl radicals	[101]

* The antioxidant properties of silver nanoparticles were studied along with the antidiabetic effect.

**Table 3:** Animal models to evaluate the antidiabetic activity of AgNPs.

In Vivo	Strain/Drug Dose	Fundamentals	Reference
Diabetic mice induced with alloxan	Swiss albino mice/single i.p. injection of alloxan 150 mg/kg; Albino mice/ single i.p. injection 35 mg/kg; Albino mice/single i.p. injection 180 mg/kg.	Intraperitoneal application of alloxan in mice causes hyperglycemia and induces diabetes.	[96,102,103,104]
Diabetic rats induced with alloxan	Wistar albino rats/ single i.p. injection of alloxan 200 mg/kg; Wistar rats/ single i.p. injection 150 mg/kg.	Alloxan injection damages rat pancreas and induces diabetes.	[105,106,107]
Diabetic mice induced with streptozotocin (STZ)	BALB/c mice/ single i.p. injection of STZ 45 or 50 mg/kg; BALB/c mice with high-fat diet and two doses of STZ 100 mg/kg; Swiss albino mice/single i.p. injection of STZ 65 mg/kg.	Streptozotocin injection damages mouse pancreas and induces diabetes.	[80,88,97,108,109]
Diabetic rats induced with streptozotocin	Wistar rats/single i.p. injection of streptozotocin dose (40, 50, 55 or 60 mg/kg); A high-fat diet during 7 weeks followed by an i.p. low dose of streptozotocin (30 mg/kg).	Streptozotocin injection damages rat pancreas and induces diabetes.	[110,111,112,113,114,115,116]
Zebra fish	Immersion of adult zebrafish (*Danio rerio)* in 111 mM glucose solution for 14 days.	Immersion in a glucose solution resulted in a sustained hyperglycemic state.	[93]

**Table 4:** In vitro antidiabetic effects of AgNPs biosynthesized with plants.

Plant	Nanoparticle Size	In Vitro Method	Concentration	Results	Reference
*Gracelliaria edulis* and *syringodium isoetifolium*	71 and 110 nm	α-amylase inhibition Glucose difusion inhibition test	Four concentrations ranging from 100 to 400 μg/mL	*Gracillaria Edulis* AgNPs were more effective for amylase inhibition (68.75–98.75%) than syringodium isoetifolium AgNPs (25.35–77.25%). *Gracillaria Edulis* AgNPs were more effective (58.75–78.75%) inhibiting glucose diffusion than syringodium isoetifolium AgNPs (10.35–45.25%).	[74]
*Ocimum basilicum*, and *Ocimum sanctum* (L.)	Size range of 17.0 ± 8.94 nm for *O. basilicum* AgNps; 15.0 ± 12.34 nm for *O. sanctum* AgNps, and 17.0 ± 8.44 nm for the combination *O. sanctum* combined with *O. basilicum* AgNps	α-amylase and α-glucosidase inhibition	α-amylase assay: 3 mg/mL α-glucosidase assay: 0.3 mg/mL	The AgNPs derived from *O. sanctum* (AgNPs OS) and *O. basilicum* (AgNPs OB) displayed an inhibitory effect on α-amylase: 59.57 ± 3.72% and 59.79 ± 6.91, respectively. AgNPs OS inhibited α -glucosidase at 89.31 ± 5.32%, while AgNPs OB inhibited 79.74 ± 9.51%.	[121]
*Punica granatum*	Particle size ranging from approximately 35 to 60 nm. Average size 48 nm Z potential −26.6 mV	α-Amylase and α-glucosidase inhibition. ABTS and DPPH assays	20–100 μg/mL	AgNPs inhibited α-amylase and α-glucosidase (IC_50_: 65.2 and 53.8 μg/mL, respectively). ABTS (IC_50_ = 52.2 μg/mL) and DPPH (IC_50_ = 67.1 μg/mL) antioxidant activity.	[99]
*Melia azedarach*	14–20 nm. Mean size: 17.75 ± 1.26 nm	α-Amylase and α-glucosidase inhibition DPPH and ABTS assay	200 and 400 μg/mL	AgNPs (400 μg/mL) demonstrated high antidiabetic efficacy as measured by α-amylase (85.75%) and α-glucosidase (80.33%) inhibition assays. Antioxidant activity by DPPH: (63.83%) and ABTS (63.61%) radical scavenging assays.	[75]
*Ananas comosus* (L.)	Not specified	α-glucosidase inhibition DPPH and ABTS assays	Enzyme inhibition: 0.008–1.000 μg/mL (10 μg/mL serially diluted) DPPH and ABTS assays: 25–100 μg/mL	AgNPs at concentrations of 0.063, 0.125, 0.250, 0.500, and 1.000 μg/mL showed a 100% α-glucosidase inhibition DPPH scavenging activity: 27.23% to 43.41%. Scavenging activity in ABTS: 9.18–13.32%.	[76]
*Ipomoea batatas* (L.) Lam peels, varieties Korean red skin, and Korean pumpkin	Not specified	α-glucosidase inhibition DPPH, ABTS, and NOX assays Reducing power assay	0.25–1.00 μg/mL	α-glucosidase inhibition: Korean red skin AgNPs: IC_50_ = 0.36 μg/mL; Korean pumpkin AgNPs IC_50_ = 0.77 μg/mL. The antioxidant activity was higher in AgNPs from Korean pumpkin skin than Korean red skin.	[100]
*Phaseolus vulgaris*	78.02 nm	α-glucosidase inhibition DPPH, ABTS, NOX, reducing power assays	1, 5, and 10 µg/mL	Glucosidase inhibition IC_50_ = 1.98 μg/mL. A reasonable antioxidant activity.	[122]
*Pisum sativum* L.	10–25 nm	α-glucosidase inhibition DPPH assay	1.0–10 µg/mL	α-glucosidase (95.29% inhibition at 10 µg/mL and IC_50_ = 2.10 µg/mL. Moderate antioxidant activity (50.17% reduction of DPPH at 100 μg/mL).	[123]
*P. americana, Beta vulgaris*, and *Arachis hypogaea* shell	Not specified	α-glucosidase inhibition DPPH ABTS Reducing power	1.0–5.0 µg/mL 25, 50 and 100 µg/mL	AgNPs of *Arachis hypogaea* shell exhibited a higher α-glucosidase inhibition IC_50_ value (1.68 μg/mL) than the other two tested AgNPs. The three generated AgNPs showed a moderate antioxidant activity.	[124]
*Equisetum arvense* plants	170.5 nm	α-glucosidase inhibition DPPH, ABTS, NOX, and reducing power assays	0.5–2.5 µg/mL 25, 50 and 100 µg/mL	α-glucosidase inhibition ranged from 20 to 95.77%. The IC_50_ value was estimated as 1.73 μg/mL. High antioxidant effect	[125]
*Leucosidea sericea*	7.8, 2.9, and 3.3 nm	α-Amylase and α-glucosidase inhibition ABTS and FRAP assays	2.5–200 µg/mL	AgNPs made with total extract and two different extract fractions. AgNPs exhibited α-amylase inhibition range: IC_50_ = 13.24–19.13 µg/mL. AgNPs α-glucosidase inhibition: IC_50_ = 8.75–21.48 µg/mL. AgNPs showed comparable or significantly lower antioxidant activity than those of the intact extract fractions.	[120]
* Silybum marianum *	18.12 and 13.20 nm	α-Amylase and α-glucosidase inhibition *ABTS, FRAP, DPPH, TAC,* and *TRP*	Not specified	AgNPs from plant and seeds α-amylase inhibition: 25.36% and 26.78%, respectively AgNPs exhibited lower enzyme inhibition and antioxidant activities than those of the extracts.	[72]
* Ficus palmata *	9.26 nm	α-Amylase and α-glucosidase inhibition	10.0–90.0 µg/mL	α-amylase inhibition IC_50_ = 27 μg/mL α-glucosidase inhibition IC_50_ = 32 μg/mL	[126]
* Annona muricata *	86.78 nm	α-Amylase and α-glucosidase inhibition DPPH and ABTS assays	Not specified	AgNPs displayed strong activities against α-amylase (IC_50_ = 0.90 μg/mL), α-glucosidase (IC_50_ =3.32 μg/mL), DPPH (IC_50_ = 51.80 μg/mL), and ABTS (IC_50_ = 30.78 μg/mL)	[127]
* Cleome viscosa *	5–50 nm	α-Amylase and α-glucosidase inhibition Assay of non-enzymatic glycosylation of hemoglobin (HbA1c) DPPH, ABTS, H_2_O_2_, Phosphomolybdenum method, and reducing power ability assays	20.0–100.0 µg/mL	One type of the AgNPs synthesized exhibited IC_50_: 21.92 ± 1.74 and 21.76 ± 1.91 µg/mL of α-Amylase and α-glucosidase inhibition, respectively. Inhibition of enzymatic glycosylation of hemoglobin, IC_50_ = 159.31 ± 9.9 µg/mL. AgNPs displayed good antioxidant activity	[85]
* Odontosoria chinensis * (L.)	22.3–48.2 nm	α-amylase inhibition	50, 100, 150, 200 and 250 µL/mL	21.53–51.85% of α-amylase inhibition	[128]
*Heritiera fomes* and *Sonneratia apetala*	20–30 nm and 70–100 nm	α-amylase inhibition	100–500 µg/mL	280.39 and 273.48 μg/mL for AgNPs from bark and leaf extracts, respectively	[43]
* Vitis vinifera *	20–35 nm	α-amylase and α-glucosidase inhibition DPPH and ABTS assays	20, 40, 60, 80, and 100 µg/mL	Inhibition of α-amylase (IC_50_, 60.2 ± 2.15 μg/mL) and α-glucosidase (IC_50_, 62.5 ± 2.75 μg/mL). AgNPs exhibited significant free radical scavenging activity, mainly DPPH radical (IC_50_, 50.0 ± 2.25 μg/mL) and ABTS radical (IC_50_, 38.46 ± 1.14 μg/mL).	[78]
* Morus macroura *	22.93 nm	α-amylase and α-glucosidase inhibition	Not specified	α-amylase inhibition 67.77 ± 3.29% and α-glucosidase inhibition 35.83 ± 2.40%. Anti-glycation activity (37.68 ± 3.34%) against pentosidine-like advanced glycation end products (AGEs) and 67.87 ± 2.99% against vesperlysine-like AGEs.	[129]
* Zinger officinale *	Not reported	α-amylase and α-glucosidase inhibition	10 to 50 µg/mL	At the highest concentration, the α-amylase inhibitory assay and the β-glucosidase inhibitory assay showed approximately 78% and 80% inhibition.	[79]
* Allium sativum *	10–30 nm	α-amylase and α-glucosidase inhibition Utilization of Glucose by the L-6 cell line. Glucose production in HepG2. DPPH radical scavenging activity	20 to 100 µg/mL	AgNPs inhibited significally higher α-amylase and α-glucosidase compared to control acarbose. Glucose uptake level from 28.9 to 41.5%. AgNPs (50 μg/mL) surpassed the glucose uptake ability (32.4%) compared to the control metformin (28.8%). AgNPs significantly inhibited glucose production over the control at all the tested concentrations. AgNPs exhibited the antioxidant activity of 31% to 63% with an average IC_50_ value of 61.81 ± 19.4. AgNPs exhibited lower scavenging activity of DPPH than the standard (IC_50_ value 32.63 ± 14.8).	[87]
* Brassica oleracea *	276.35 nm	α-glucosidase inhibition DPPH, ABTS, NOX, and reducing power assays	1.0–5.0 μg/mL	At 2.5 μg/mL AgNPs displayed 80.44% inhibition	[130]
* Korean Ueong *	170.3 nm	α-glucosidase inhibition	1.0–100.0 μg/mL	α-glucosidase with a maximum inhibition value of 95.41% at 5.0 µg/mL and more than 86% inhibition at 2.5 µg/mL and the estimated IC_50_ value was found to be 0.653 µg/mL	[131]
* Rosa indica * L.	18, 12, and 770 nm	α-amylase and α-glucosidase inhibition DPPH assay	10, 25, 50, 75, and 100 µg/mL)	IC_50_ values of α-amylase and α-glycosidase being 50, 50, and 75 µg/mL for ethanolic, acetone and aqueous extract synthetized AgNPs. DPPH scavenging was well, higher with AgNPs synthesized with ethanolic and acetone extract.	[132]
* Kigelia africana *	25–35 nm	PPARγ expression (semiquantitative Real Time-PCR) in RINm5F insulinoma cells	25 and 50 µg/mL	AgNPs upregulated PPARγ expression	[92]
* A. nilotica *	20–50 nm	α-glucosidase inhibition DPPH assay	50 and 250 µg/mL	Silver nitrate at 0.1 M and 3 mM were used to synthesize AgNPs, α-glucosidase inhibition range: 47.87–73.93%	[133]
* Fagonia cretica *	20 to 50 nm	α-amylase, α-glucosidase inhibition DPPH assay	62–1000 µg/mL	α-amylase inhibition 51.10 to 83.53%. α-glucosidase inhibition 39.50 81.74%.	[80]
* Solanum khasianum *	15.96 nm	α-amylase inhibition	50–250 μg/mL	Maximum inhibition of 79.56%	[134]
* Achillea maritima subsp. Marítima *	14.13 to 21.26 nm	α-amylase, α-glucosidase inhibition DPPH assay	20–120 μg/mL	α-amylase IC_50_ = 64.9 μg/mL. α-glucosidase IC_50_ = 41.6 μg/mL. DPPH assay IC_50_ = 41.6 μg/mL Strong antioxidant activity	[135]
* Aeonium haworthii *	35–55 nm	α-amylase inhibition DPPH assay	20–120 μg/mL	α-amylase inhibition IC_50_ = 62.84 μg/mL DPPH antioxidant assay: IC_50_ = 0.044 mg/mL	[136]
* Cleome brachycarpa *	20 to 80 nm	α-amylase inhibition DPPH assay Reducing power assay	0.3–1.5 μg/mL	Higher α-amylase inhibition than control acarbose Similar antioxidant activity to butylhydroxytoluene	[137]
* Azadirachta indica *	34.43 nm	Glucose uptake by yeast α-amylase inhibition	10, 20, 40, 80, 100 μg/mL	AgNPs showed the highest activity (75.0%) glucose uptake by yeast. The alpha-amylase assay, AgNPs exhibited the maximum activity of 73.85%	[88]
* Taraxacum officinale *	45–55 nm	α-glucosidase inhibition	100, 300, and 600 μg/mL	α-glucosidase enzyme inhibitory effect (88.37%) in comparison with controls C-AgNPs1 (61.7%) and C-AgNPs2 (50.5%).	[138]
* Cymodocea serrulata * (R.Br.) Asch. & Magnus	60 and 69 nm	α-amylase, α-glucosidase inhibition DPPH, hydroxyl scavenging activity, and ABTS assays	25–125 μg/mL	AgNPs exhibited α-amylase and α-glucosidase inhibition of 13.56–57.31% and 15.78–54.5%, respectively. AgNPs strongly impacted free radical scavenging against DPPH, H_2_O_2_ radicals, and the ABTS test.	[139]
* Ageratum conyzoides *	30–90 nm	α-amylase inhibition DPPH, FRAP, hydrogen peroxide scavenging assays	3.12–100 μg/mL	α-amylase inhibition: IC_50_ = 21.52 μg/mL. Better antioxidant property than extract: DPPH 87.86%, FRAP 85.95%, and H_2_O_2_ assay 90.11%	[140]
* Solanum tuberosum * and *Coriander sativum*	65 nm for potato peels extract and 70 nm for coriander stems extract	α-amylase inhibition DPPH assay	Not specified	AgNPs from potato peel extracts inhibited more than positive control (86.72 ± 0.19%). AgNPs from the coriander stem extracts showed 85 ± 0.98% as compared to control. Strong antioxidant activity, higher than their respective extracts.	[141]
* Salacia oblonga *	99.8 nm	α-amylase inhibition DPPH assay, reducing power capacity, and hydroxyl radical scavenging assay (HRSA)	20–100 μg/mL	EC_50_ for α-amylase inhibition = 58.38 μg/mL, IC_50_ DPPH assay: 80.64 μg/mL, reducing power capacity: 81.09 μg/mL, nitric oxide 96.58 μg/mL, and hydroxyl 58.38 μg/mL radical scavenging activities.	[101]
* Calotropis procera *	23.8 nm	α-amylase inhibition Hydrogen peroxide scavenging activity	Not specified	α-amylase inhibitory activity higher (36.33%) than that of metformin (1.44%) Remarkable reducing capacity of AgNPs compared to the ascorbic acid.	[142]
* Aconitum lycoctonum L. (Ranunculaceae) *	67 nm	α-amylase inhibition FRAP and DPPH assays	10–30 mg/mL	The highest concentration achieved 59.12% inhibition. Good antioxidant potential: the highest value of FRAP (50.47%) was detected at a concentration of 90 ppm and a DPPH scavenging activity of 69.63% was detected at a concentration of 20 μg/mL of AgNPs.	[143]
* Cucumis melo * L.	66.7–92.3	α-amylase and α-glucosidase inhibition	20–100 μg/mL	AgNPs showed the highest inhibition activity on both enzymes at the 100 µg/mL concentration	[144]
* Thymus Vulgaris *	44.6 nm	α-amylase inhibition DPPH assay	250–1000 μg/mL	Inhibitory activity was observed from 50.67% to 82.57%. The antioxidant activity of AgNPs showed 92% inhibition at the concentration of at 1000 μg/mL.	[145]
* Duabanga grandiflora *	99.72 nm	α-amylase inhibition DDPH assay	10–1000 μg/mL	α-Amylase inhibitory activity: IC_50_ = 162.11 μg/mL	[146]
* Salvia blepharophylla * and *Salvia greggii*	52.4 and 62.5 nm	α-amylase inhibition DDPH assay	20–100 μg/mL	α-amylase inhibition of* Salvia blepharophylla* AgNPs: 35.4% and 86.5%; for *Salvia greggii AgNPs*: 29% and 80.5% Higher antioxidant activity than standard	[147]
* Drymaria cordata *	5–100 nm	α-amylase and α-glucosidase inhibition DPPH and ABTS assays	25–1000 μg/mL	α-amylase inhibition: 68.92 ± 0.16%; α-glucosidase inhibition: 66.79 ± 0.08%. AgNPs exhibit better scavenging activity than *D. cordata* extracts.	[148]
* B. aegyptiaca *	10.352 nm	Glucose Uptake Assay in C2C12 cells Insulin secretion in Min6 cells	6.25–100 μg/mL	Glucose increase uptake: 156.00% compared to control. Insulin enhanced secretion 3.92-fold compared to control.	[91]
* Podocarpus macrophyllus *	13 nm	α-amylase inhibition DPPH assay	200–1000 μg/mL	α-amylase inhibition 92.7% at 1000 μg/mL 90% free radical scavenging	[149]
* Ipomoea aquatica *	36.27 nm	α-amylase inhibition DPPH assay	Not specified	α-amylase inhibition = 78.55%. Significant antioxidant activity, surpassing standard ascorbic acid.	[150]
* Cymbopogon citratus *	135.41	α-amylase and α-glucosidase inhibition DPPH, ABTS, and TRP assays	10–50 μg/mL	α-amylase inhibition IC_50_ = 34.81 μg/mL α-glucosidase inhibition IC_50_ = 20.84 μg/mL Strong antioxidant activity at higher concentration	[73]
* Melia azedarac *	20–30 nm	α-amylase inhibition DPPH assay	Not specified	α-amylase inhibition: 80.33%. Strong free radical scavenging properties.	[98]
* Berberis lyceum *	11.02 nm	α-amylase and α-glucosidase inhibition DPPH assay	50–250 μg/mL	α-amylase inhibition: 78 ± 0.38% α-glucosidase inhibition: 88 ± 0.58%. α-amylase and α-glucosidase inhibition were similar to that of acarbose, also higher than the extract. Higher antioxidant activity than that of the extract.	[103]

**Table 5:** Antidiabetic effects of AgNPs biosynthesized in animal models.

Origin	Nanoparticle Size	Animal Model (Species, Strain, Sex, Age, Weight)	Dose and Time of AgNPs	Results	Reference
* Eysenhardtia polystachya *	10–12 nm	Glucose-induced diabetic zebrafish.	5 and 10 μg/mL. 14 days.	Diminution of hyperglycemia improved hyperlipidemia.	[93]
*E. phyllantus*	30, 45, and 65 nm	Albino mice, 60 days old (male or female), weighting 18−21 g Diabetes induction with alloxan	150 and 300 mM for 15 days.	A significant decrease in the glucose level and significant recovery in the liver and kidney.	[102]
*Cucumis sativus*	27–97 nm	Adult albino mice, 4 and 6 weeks, weight approximately 25 g. Diabetes induction with alloxan	A 5% ointment: 50 mg of silver nanoparticles into 1000 mg of pure vaseline. 15 days.	Nanoparticles ointment treated mice showed significant wound contraction at day 15 as compared to control.	[104]
*Azadirachta indica*	Not reported	Mature male Swiss albino mice, weight: 30–35 g; average age 8 weeks. Diabetes induction with alloxan	AgNPs 100 mg/kg; AgNPs 100 mg/kg + glibenclamide. 28 days.	Improvement in the body weight and blood glucose level. Significant regeneration in the histomorphology of the kidney, liver’s central vein, and islets of Langerhans Radical scavenging activity.	[96]
*Berberis lyceum*	11.02 nm	Diabetic induced by alloxan, Swiss albino mice weighing 38.6 ± 3.0 g. Average age: 8 weeks	200 mg/kg. 28 consecutive days.	AgNPs decreased in blood glucose.	[103]
*Phagnalon niveum*	12 to 28 nm. Average: 21 nm.	8-week-old Wistar rats weighing 140–150 g. Alloxan-induced diabetes.	10 mg/kg of body weight for 21 days.	A significant reduction in blood glucose levels and an increase in body weight, as well as a remarkable improvement in lipid, liver, and kidney profiles, were noticed.	[107]
* Solanum nigrum *	4–25 nm	Male Wistar albino rats aged more than 8 weeks (140–160 g body weight). Diabetes induction by single dose of alloxan	AgNPs 10 mg/kg for 21 days.	Reduced the blood glucose level over the period of treatment. Improved the dyslipidemic condition and body weight.	[105]
* Eryngium* *thyrsoideum Boiss*	10 and 56 nm	Male Wistar rats weighing 180–200 g. Alloxan induced diabetes	2.5 mg/kg for 14 consecutive days.	Silver nanoparticles decreased significantly liver enzyme levels including alanine aminotransferase (ALT) and aspartate aminotransferase (AST) and may exert protective effects on liver damage.	[106]
*Camellia sinensis*	15.954 nm	Male Swiss albino mice (20–25 g) Diabetes induction with streptozotocin	100 mg/kg/ bw/day. 14 days.	AgNPs reduced blood glucose, total cholesterol, triglyceride, low-density lipoprotein (LDL) and creatinine levels.	[109]
*Fagonia cretica*	29.39 nm	Male Balb/C albino mice that were 6 weeks old and weighed between 25 and 35 g Diabetes induction with streptozotocin	200 mg/kg body weight. 21 days.	Significant weight gain and a decrease in all biochemical markers (blood glucose, pancreas panel, liver function panel, renal function panel, and lipid profile).	[80]
*Azadirachta indica*	34.43 nm	Adult BALB/C mice weighing 25–30 g and 5 weeks old. Diabetes induction: one dose (50 mg/kg body weight) of streptozotocin i.p.	10–40 mg/kg Treatment for 30 days.	A significant decrease in blood glucose level. Diabetic mice treated with different doses of AI-AgNPs revealed regeneration of islet cells in pancreas. The diabetic group of mice treated with 40 mg/kg b/w showed a histological appearance that was comparable to the normal control group.	[88]
*Thymus serpyllum*	Average: 42 nm	4-week-old male BALB/c mice. Mice were fed a high-fat diet and low doses of streptozotocin	5 and 10 mg per kg of body weight for 28 days.	A 10 mg/kg dose increases the expression of AMP-activated protein kinase (AMPK) and insulin receptor substrate 1 (IRS1), enhancing glucose uptake in cells.	[97]
*Tribulus terrestris*	22 nm	5-week-old BALB/C mice, weight: 25–30 g. Diabetes induction with streptozotocin.	10, 20, 30, or 40 mg/kg for 30 days.	Treated groups displayed histological improvements in pancreas and liver, also blood glucose levels dropped in a dose-dependent manner.	[108]
*Momordica charantia*	Less than 100 nm	Male Wistar rats, weighing between 180 and 200 g. Diabetes induction with a single intraperitoneal dose of streptozotocin.	800, 1000, and 2000 mg/kg for 14 weeks.	AgNPs showed potent anti-hyperglycemic properties and improved different entanglements of diabetes.	[111]
*Psidium guajava*	52.12–65.02 nm	Both sexes of 160–200 g Wistar. Streptozotocin-induced diabetic rats.	200 and 400 mg/kg for 21 days.	Decrease in the blood glucose level, preventing subsequent weight loss and ameliorating lipid profile parameters. Improvements in pancreas and liver cells	[113]
*Phragmanthera austroarabica*	13 nm	Male Wistar rats 130 and 170 g. High-fat diet for 7 weeks and a low streptozotocin injection	200 mg/kg/day, p.o. 4 weeks.	AgNPs decreased insulin, serum glucose, leptin, and Homeostatic Model Assessment of Insulin Resistance (HOMA-IR).	[114]
*Salvia Sclarea*	Average size range 40 ± 5 nm	Adult male Wistar rats with weights between 100 and 150 Diabetes induction with streptozotocin	10 mg/kg/day, intraperitoneal. 16 weeks.	AgNPs led to a significant rise in glutathione, superoxide dismutase, glutathione peroxidase, and catalase enzyme levels and decreased malondialdehyde levels in the treated group. AgNPs attenuated hyperglycemia induced oxidative stress, inflammation by reducing TNF-α, IL-1β and PKC-β in renal cells of diabetic rats	[116]
*Ziziphora clinopodioides*	Below 100 nm	Streptozotocin-induced Wistar diabetic male rats	50–400 μg/kg 20 days of treatment.	AgNPs reduced the fasting blood glucose levels compared to the diabetic group.	[115]
*Rumex hymenosepalus*	9 ± 3 nm	Streptozotocin-induced Male rats of the Wistar strain, weighing 150 ± 15 g.	150 μg/kg. 9 days.	Treatment during 9 days with AgNPs decreased 50% the blood glucose in diabetic rats. The glucose tolerance test showed that in diabetic rats treated with AgNPs, there was a minimal increase in blood glucose.	[110]
*Lawsonia inermis*	14.9 nm	Male Wistar rats, weighing between 180 and 200 g Diabetes induced with streptozotocin	200 mg/kg. 14 days.	Potent hypoglycemic activity compared to the extract group	[112]
